# The impact of the first COVID-19 lockdown in the UK for doctoral and early career researchers

**DOI:** 10.1007/s10734-021-00795-4

**Published:** 2021-12-14

**Authors:** Patricia C. Jackman, Rebecca Sanderson, Tandy J. Haughey, Caroline E. Brett, Naomi White, Amy Zile, Katie Tyrrell, Nicola C. Byrom

**Affiliations:** 1 School of Sport and Exercise Science, University of Lincoln, Lincoln, UK; 2 Lincoln Higher Education Research Institute, University of Lincoln, Lincoln, UK; 3 Sport and Exercise Sciences Research Institute, Ulster University, Belfast, UK; 4 School of Psychology, Liverpool John Moores University, Liverpool, UK; 5 Institute of Health and Wellbeing, University of Glasgow, Glasgow, UK; 6 School of Health Sciences, University of East Anglia, Norwich, UK; 7 Research Directorate, University of Suffolk, Suffolk, UK; 8 Department of Psychology, Institute of Psychiatry, Psychology and Neurosciences, King’s College London, London, UK

**Keywords:** Mental health, Isolation, Pandemic, Psychological wellbeing, University

## Abstract

Doctoral researchers and early career researchers (ECRs) are crucial to producing scientific advancements and represent the future of academic leadership. Their research endeavours were changed radically by lockdowns in response to the COVID-19 pandemic. The aim of this study was to explore the perceived benefits and challenges of the national lockdown in the UK from the perspective of doctoral researchers and ECRs. We present analysis of qualitative survey data from 1,142 doctoral researchers and ECRs on their experiences of the first UK lockdown collected from April 16, 2020–May 14, 2020. Our findings suggest considerable heterogeneity in how the pandemic impacted this key group of academic workers. Challenges arising from the lockdown largely cohered around a poor work environment, limited access to resources, perceptions of pressure, and negative psychological outcomes. Conversely, respondents also highlighted several benefits in the early stages of the pandemic, with the change to working from home creating more time, resulting in greater productivity and a better work-life balance. Collectively, findings indicate the importance of considering the personal circumstances and needs of individual researchers. We discuss the implications for support these researchers require to rebuild their careers in the wake of the initial disruption.

## Introduction

The global COVID-19 pandemic has caused rapid and unprecedented changes in how universities operate around the world. This initial shift occurred for the higher education (HE) sector in the UK at the time of the first national lockdown in March 2020, when universities physically closed their premises and asked the vast majority of employees to work from home to curb the spread of the virus. Eighteen months on, the vaccine roll-out offer some hope of a return to normality, but many fear the impact of the virus might need to be tolerated on an indefinite basis (Kissler et al., 2020). As the HE sector considers the future work environment, many have suggested this unprecedented period of change offers an opportunity to “reset” the scientific establishment and how the next generation of researchers are supported by universities, funders, and the public (Gibson et al., 2020). Supporting doctoral researchers and early career researchers (ECRs) should be a key priority for HE institutions, because a lack of support could result in the pandemic having an even greater negative impact on the scientific community. Doctoral researchers and ECRs are vital to economic growth, innovation, and scientific knowledge (OECD, 2019) and represent the research leaders of the future. Furthermore, as a substantial proportion of this cohort are likely to remain in academia (Woolston, 2019), investing in the futures of doctoral researchers and ECRs is vital for protecting the education of future generations of university students (Greener, 2021).

In the early stages of the pandemic, researchers urged their community to take this opportunity to re-organise priorities, focus on collective rather than individual goals, and pay more attention to mentoring and supporting students (Corbera et al., 2020). Inherent in this call was a recognition of the inequalities that the lockdown accentuated (Tatham, 2020; Witteman et al., 2021). Higher education commentators have long talked of reimagining the future of university, freed from the binds of neoliberal excess (Izak et al., 2017). Against this backdrop, some have warned that we must not wait to see what happens (or does not), but take informed steps to re-shape our work environment (Watermeyer et al., 2021).

Prior to the pandemic, there were substantive concerns about the wellbeing of doctoral researchers and ECRs (Metcalfe et al., 2018), with evidence indicating a high prevalence of mental distress (Evans et al., 2018; Levecque et al., 2017; Panger et al., 2014). These researchers constitute the most vulnerable group in our institutions, lacking a career track record or job security. Furthermore, they are often the first to suffer from the stress that has befallen this system (e.g., from the emergence of ‘steady state science’—Cozzens, 1990). Doctoral researchers face role ambiguity and conflict, with high work demands for relatively low reward or support (Metcalfe et al., 2018; Schmidt & Hansson, 2018). This, combined with a lack of positive feedback on progress (Metcalfe et al., 2018), may contribute to high levels of self-deprecation (Byrom et al., 2020). These challenges are accentuated by a culture of poor work-life balance, poor supervisory relationships, financial and career concerns, and social isolation (Byrom et al., 2020; Metcalfe et al., 2018).

Low career confidence is a substantive contributing factor to the experience of distress among doctoral researchers (Byrom et al., 2020). This lack of confidence is unsurprising as there is a substantive mismatch between doctoral researchers’ expectations and the harsh reality of building careers within HE (Cornell, 2020). Current employment conditions for this community do little to improve the confidence of ECRs, with most facing many years of short-term contracts and continual job insecurity (Dorenkamp & Süß, 2017). During the pandemic, these existing problems may have been exacerbated by the time-constrained nature of fixed-term contracts and doctoral programs, as delays—or stoppages—in research caused by lockdowns could jeopardise their research and career development (Paula, 2020). As such, the HE sector has a responsibility to remain aware of the profound impact the lockdowns have had on doctoral researchers and ECRs (Corbera et al., 2020).

Therefore, the purpose of this study was to explore qualitatively the impact of the COVID-19 lockdown on doctoral researchers and ECRs in the UK. More specifically, we sought to address two research questions: (RQ1) what have been the benefits, if any, of the COVID-19 lockdown for doctoral researchers and ECRs?; and (RQ2) what have been the most challenging aspects of the COVID-19 lockdown for doctoral researchers and ECRs? By doing so, the study aimed to understand what the transition to pandemic-constrained research environments can tell us about the experience of doctoral researchers and ECRs more generally, and identify whether there are insights that can improve the working experience for this community as we consider the research landscape and seek to ensure that the creativity, expertise, and ideas of the next generation of researchers are not lost. In turn, findings could generate recommendations that help academic leaders improve support for this community as the sector continues to navigate the pandemic and plan for a post-pandemic world. While this study focuses on the UK, the UK is recognised as one of the driving forces of an increasingly globalised knowledge economy and is among the leading contributors to international scientific collaborations (Gui et al., 2019). Therefore, evidence generated in the UK will be relevant to the international research community.

## Methods

### Participants and sampling

The study was registered with the King's College London ethics board; MRA—19/20 – 18,347. During a 4-week period (16th of April, 2020–14th of May, 2020) in the first national UK lockdown, doctoral researchers and ECRs in the UK were invited to complete an online survey. Invitations were disseminated through social media and communication channels supported by SMaRteN (Student Mental Health Research Network), the UK Research and Innovation (UKRI)-funded student mental health research network, and Vitae, a non-profit programme working with universities to support the professional development of researchers. Invitations to complete the survey were also circulated by funding councils and universities. The invites contained a hyperlink that directed prospective participants to an online survey hosted on Qualtrics.

A total of 5,902 researchers participated in the survey, which contained quantitative^1^ and qualitative measures. For the current study, we report findings from the qualitative responses provided by a stratified random sample (*N* = 1,142), with the quantitative findings from this survey reported elsewhere (Byrom, 2020). This sample size was selected to enable analysis of a manageable volume of qualitative data and was deemed appropriate based on the concept of information power (Malterud et al., 2016). Stratified sampling ensured representation from all ethnic backgrounds and academic areas. Additionally, responses were strategically sampled from individuals who identified their gender as non-binary, to ensure that this small proportion of the overall response was not lost in sampling. Doctoral researchers in both our full survey (Byrom, 2020) and current study sample were distributed relatively equally between their 1st, 2nd, and 3rd year of study, with slightly fewer respondents from 4th year or beyond. The majority (62%) of early career researchers had been working in research for five years or less.

## Materials

Qualitative surveys can generate rich data and are well-suited to research that seeks to collect a wide range of perspectives (Braun et al., 2021). Respondents provided qualitative responses to two questions as part of a larger mixed methods survey about their experience of the lockdown, with the survey completed online and taking approximately 20 min to complete (Byrom, 2020). In the current paper, we report analysis of responses to two open-ended questions: (1) have there been any benefits to the COVID-19 pandemic for your work?; and (2) what have been the most challenging aspects of the COVID-19 pandemic for your work?

### Data analysis

Embracing a co-creation strategy, the research team involved a collaboration between doctoral researchers (AZ, KT) and ECRs (PJ, RS, TH, CB, NW). Co-creation has multiple benefits, helping to ensure that analysis is grounded in the stakeholder perspective and offering an opportunity to create more critical and in-depth interpretations (Gibson et al., 2017). Based on our personal experiences of the COVID-19 lockdown as researchers in the UK, we held some “insider knowledge”. Although this aspect of our positionality aided our interpretations, we needed to remain individually and collectively reflexive throughout our analysis (Lazard & McAvoy, 2020) to ensure our experiences were not amalgamated with the participants’.

Data from the included participants were divided into five clusters, with each cluster analysed by one researcher. The remaining three researchers sampled from across the clusters, such that approximately 50% of responses were analysed by two researchers. Our analysis employed an inductive, data-driven approach, therefore focusing on understanding any benefits of the COVID-19 lockdown, and what constituted challenges while working from home through the pandemic without a pre-existing theoretical framework. Following the steps for thematic analysis (Braun et al., 2016), each coder initially familiarised themselves with the survey responses for their allocated cluster and produced codes to represent each response. Given our ambition to create a broad depiction of the researchers’ experiences, coding was completed at a semantic level (Braun et al., 2016). We then revisited the codes developed and proposed preliminary themes. The first author, in collaboration with the research team, then refined the final themes and reviewed the analysis to identify connections between themes within the analysis (Maxwell, 2012). After establishing our codes and themes, we reviewed our analysis and conducted subgroup analyses by comparing findings based on: (1) researcher status (i.e. doctoral researcher [DR] or post-doctoral ECR [ECR]); (2) academic area (i.e. social sciences [SS], science, technology, engineering, and mathematics [STEM], medical sciences [MS], or arts and humanities [AH]); and (3) caring responsibilities. A description of the analysis was written (PJ) and then critically reviewed by all researchers, to encourage further reflexivity (Braun & Clarke, 2019). In the written report of our analysis that follows, at times, we have “cleaned” quotes (punctuated and corrected typographical errors) for ease of reading. Themes are italicised in the text, with information added on participants’ researcher status and academic area for illustrative quotes.

## Results

Of the total sample analysed (Table 1), 955 identified challenges, while 492 described benefits. There was substantive overlap between benefits and challenges, with respondents experiencing the same aspects of the lockdown differently. This heterogeneity emphasises the need for institutions to recognise the diversity of experiences, priorities, and personal circumstances. The overarching domain summaries, perceived challenges of the lockdown and benefits of the lockdown, are next presented.

**Table 1:** Demographic characteristics of sample

Category	Sub-category	Full survey^1^ (*N* = 5,902)		Current study (*N* = 1,142)	
Researcher status	Doctoral researchers	4,274	72%	718	63%
	Early career researchers	1,628	28%	424	37%
Caring responsibilities		1,430	24%	331	29%
Gender^2^	Female	3,526	60%	613	54%
	Male	1,722	29%	332	29%
	Non-binary (or alternative term)	52	1%	27	2%
UK citizen		3,315	56%	609	53%
Ethnicity	White (British)	2,699	46%	391	34%
	White (other)	1,484	25%	240	21%
	Black or Black British	171	3%	53	5%
	Asian or Asian British	461	8%	88	8%
	Mixed ethnicity	194	3%	79	7%
Russell Group^3^		3,432	58%	624	55%
Funding	Research councils	1,906	32%	293	26%
	Charities	537	9%	136	12%
	Other UK government	477	8%	102	9%
	University funding	1,098	19%	214	19%
	Self-funding	759	13%	129	11%
	Other	1,125	19%	235	21%
Academic area	Medical sciences	2,321	39%	326	29%
	Science, technology engineering, and mathematics (STEM)	1,670	28%	266	23%
	Social sciences	1,322	22%	230	20%
	Arts and humanities	552	9%	289	25%

Notes: 1. Details on the full survey sample are reported elsewhere (Byrom, 2020); 2. Gender was not reported by 170 participants; 3. The Russell Group is a self-selected association of 24 public research universities in the UK

### Perceived challenges of the lockdown

**Fig. 1:**
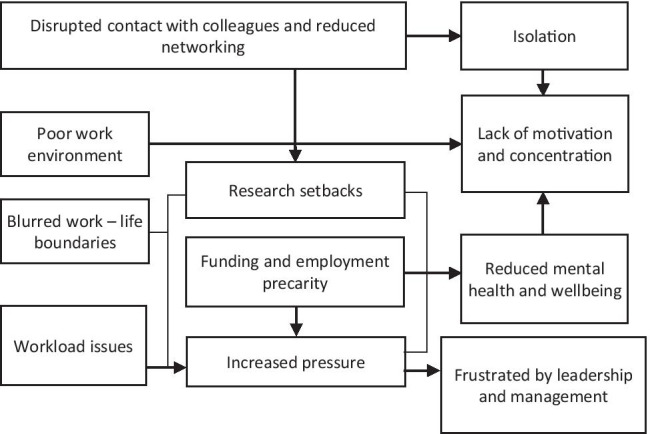
Challenges of lockdown reported by doctoral and early careers researchers

Challenges were organised into 11 interconnected themes (Fig. 1). As a result of universities closing, researchers were working from home, which, for many, was a *poor work environment*. Problems identified related to unreliable internet connectivity, small computer screens, insufficient computer screens, and other general technological problems. Many reported working in spaces not designed for work (e.g. living room, kitchen) and/or were contending with a noisy work environment due to family or neighbours. Furthermore, many felt the ergonomic set-up in their home made work more difficult and, in some cases, led to physical discomfort:

The impact of not having an appropriate workspace for me has been the most negative…I am used to having a large desk space, two screens and no distractions in my office. Now I am distracted by my partner…I cannot find a comfortable place to work and only have my laptop which has a small screen. This makes writing for me quite difficult. (DR, MS)

Finding a space that I can work at - my dining table is now my desk, my kitchen/living room/bedroom also my office. It is uncomfortable (painful) but also leaves the feeling that you can never have time away from your work*.* (ECR, SS)

As conveyed in the previous quotation, the change in working environment contributed to *blurred work-life boundaries*. By working from home, many were pushing themselves to work long hours and were worried about whether they were doing enough: “Delays, uncertainties with timelines and most of all, blurry working hours…I tend to overwork if I am at home all the time” (ECR, MS). With the requirement for social distancing and working online, many struggled with online meetings and reported a *disruption to contact with colleagues*. A desire for informal contact with colleagues and the peer support this provides was also highlighted:

Not having others around to offer support or advice when needed right there and then. This could be directly work-related, or it could be emotional support. (ECR, MS)

Lack of social interaction amongst other things has meant I'm not surrounded by the ideas I usually am. (DR, STEM)

Additionally, researchers missed external input into their work and some found it harder to obtain feedback and meet with supervisors or line managers:

The cancellation of conferences, and lack of opportunities for incidental/unplanned informal catch ups means I’ve not disseminated my research as effectively, nor received feedback as effectively. (ECR, MS)

I find it hard to have supervision remotely, I rely on being able to have direct contact with my supervisors and colleagues to have effective conversations about my work. (ECR, AH)

Many reported *isolation* as a challenge, especially doctoral researchers, as one pointed out: “Completing a PhD is already an incredibly isolating experience, COVID-19 has amplified that. I am really struggling to focus on work” (DR, MS). The *disruption to contact with colleagues* impaired opportunities for informal interactions and the peer support this offers. Some pointed out that the loss of work-related support networks led to longer-term worries about their career development: “I have also lost the support network I had during my PhD and am unsure how Covid-19 is going to impact my career” (ECR, STEM). For others, the *isolation* was connected to missing family and close friends, which often caused distress:

Not being able to travel to see my family whilst my grandad was gravely ill was a major stress factor. (DR, SS)

I am an international student (I arrived in London in January 2020). I did not have the time to build any support network before everything started. (DR, SS)

The most commonly reported challenges were captured within the theme, *research setbacks.* For those with teaching duties, the increased time required to manage the sudden move to online teaching reduced time available for research:

As a member of research staff who teaches, my time has predominantly been taken up with familiarisation with online teaching methods. This has stopped me being able to focus on my current research. (ECR, SS)

Unsurprisingly, most doctoral researchers highlighted the disruption of their research projects as a challenge, with most citing a need to pause or redesign projects, thus creating unwanted delays and concerns about progress. Worries about the impact of delays were also salient among ECRs working on fixed-term contracts. While the proportion of participants reporting research disruptions was similar across academic areas, the lockdown restrictions appeared to present some different challenges depending on the field of study. Researchers in arts and humanities outlined how the lockdown prevented access to paper documents stored in archives and libraries. As one doctoral researcher remarked, “My entire project will have to be changed this year due to lack of access to international archives. I will essentially have to start again but without a years’ worth of funding” (DR, AH). The disruption to participant access was a widespread concern for researchers in the social and medical sciences, with many reporting interruptions to data collection and cancellation of field work activities:

I was halfway through my fieldwork year working with schools, which has been cancelled and therefore I have much less research data than anticipated. (DR, SS)

Unable to collect any new data or learn from people in the lab. I was currently in the middle of being trained on electron microscopy by lab members when the lockdown was put in force. (DR, MS)

Without the ability to access lab facilities and research sites, many STEM researchers were significantly hampered, losing access to data:

I am losing an entire growing season, which disproportionately affects my PhD . . . It may only be three months, but it’s a very critical three months and I cannot grow, monitor and test my plant samples at all. (DR, STEM)

Some medical science researchers also explained that the time available for their research was reduced because they were required for clinical duties: “When I return to clinics, I have been told my protected research time will not be possible” (ECR, MS). Many researchers reported *workload issues*. Teaching staff reported difficulties with managing the increased workload that had arisen because of additional teaching load and pastoral care responsibilities:

Heavier teaching focus due to move to online requiring considerable preparation, design, increased meetings, training and learning new platforms, practice and re-build of already prepared materials. (ECR, SS)

Juggling everything as I’m a manager, lecturer and researcher. It has been an impossible few weeks, and I feel exhausted. (ECR, AH)

Some perceived discrepancies in workload and productivity related to personal circumstances, such as caring responsibilities or a commitment to supporting colleagues, which were sometimes accompanied by a sense of unfairness:

Some team members now have all the time in the world, while others are completely overwhelmed because they have to home-school children. It leads to very skewed expectations of what any one individual is capable of, and to huge delays as administrators are especially overworked. (ECR, AH)

[The] failure of support from permanent colleagues means that I have been taking on work of organising and supporting other non-permanent staff. (ECR, AH).

In particular, those with caring responsibilities commonly reported a sense that they were falling behind colleagues without childcare responsibilities, who were investing more time into career-enhancing opportunities:

I am aware of colleagues without family putting in lots of time learning new skills online, writing and reading extra papers, and developing bids, and I do not feel I can compete with this while my children are being schooled at home. (ECR, SS)

*Funding uncertainty and employment precarity *were noted, particularly among doctoral researchers and those on temporary or fixed-term contracts. Without clarity and guarantees of funding extensions for research, *research setbacks* meant serious concerns about funding, and delays without funding extensions could have real implications for publications, completion of work, and future employability. Independent of delays, many worried about future employment due to job market uncertainty:

I’m facing unemployment just at the point where my career might have taken off (2 years post-PhD), and my income will drop to zero while I'm still in debt from the costs of fees/living during my PhD. This has caused stress and anxiety - and of course there are no jobs to apply for, as everyone is freezing recruitment. (ECR, AH)

Together, *blurred work-life boundaries*, *workload issues*, *research setbacks*, and *funding and employment precarity* increased perceptions of *pressure*. One researcher commented, “Data collection planned has stopped completely. [I] worry about the impact on the project and the future being more pressured as a result” (ECR, SS). The increased perceptions of *pressure*, and the reasons underlying this, led some to feel *frustrated by leadership and management*. Many experienced unrealistic workloads and a lack of clarity around actions to take in the shift to working online. Others noted that the flurry of institutional emails and instructions about managing the pandemic and working from home were distracting:

So much conflicting and incoming information from university, news, social media, etc. about how to look after self and others, when to leave the house, that I find it hard to concentrate as my anxiety (health and general) is extremely hard at the moment. (DR, MS)

The information of the University regarding online assessments are confusing, and counter-intuitive. We receive emails for every amendment when nothing is clear. (ECR, STEM)

Some also highlighted that administrative support was reduced, which, in turn, added to their administrative duties, further exacerbating the pressure they were under. As one participant said, “Assistance for probation or admin-related documents is very limited” (ECR, SS). Many reported a lack of institutional support in transitioning to working from home, with some feeling their institutions made no meaningful acknowledgement of the challenges faced:

I have not yet had a break since the pandemic. I have been as busy as ever with added pressures on working from home and looking after relatives. I am still receiving pressure from my management to publish. (ECR, SS)

Increase in workload…university management not understanding or making meaningful recognition of challenges and increased burden of online teaching. (ECR, SS)

Many also explained that these perceptions of *pressure* were resulting in *reduced mental health and wellbeing.* Participants referred to increases in stress and anxiety, as well as general mental health concerns. Many attributed the *pressure* and uncertainty surrounding future employment as a key determinant:

The sudden stoppage of academic job recruitment and having been given no guarantees on extension of my current contract (ending in July), combined with an increased workload pressure for online teaching delivery, have taken a huge toll on my mental health. (ECR, STEM)

The main issue by far has been severely limited time and increased stress due to home schooling and basic demands of securing income and paid work. (ECR, AH)

Beyond the *pressure* surrounding funding and future job prospects, *reduced mental health and wellbeing* also stemmed from the pandemic itself, with worries about getting sick, health of family, and state of the world. Such worries contributed to a *lack of motivation and concentration*:

I am becoming “battle fatigued”, and physically and mentally tired and I am struggling to maintain the impetus to continue working on the grant application. (ECR, MS)

I find it very difficult to work for long periods of time...when worries about the future impact/safety of loved ones in the pandemic often impact my day-to-day thoughts. (ECR, AH)

A *lack of motivation and concentration* was also exacerbated by a *poor work environment* while working from home, which could make it more difficult to get into and sustain a work rhythm:

Working in a non-conducive area to do work. I have been separating university as a ‘workplace’ and home as a ‘resting place’. Having to do both at the same place is quite tricky and makes me feel less energetic, productive, and innovative. (ECR, STEM)

Distractions are at an all-time high and it’s very difficult to find quiet time to write - it’s often late at night but I’m very tired! (DR, MS)

Indeed, some researchers even begun to question the worthiness of their work in the context of the pandemic. As one pointed out, “The overarching feeling that my work is not useful and that I am not contributing anything of any importance in the midst of a global crisis” (DR, AH).

### Perceived benefits of the lockdown

**Fig. 2:**
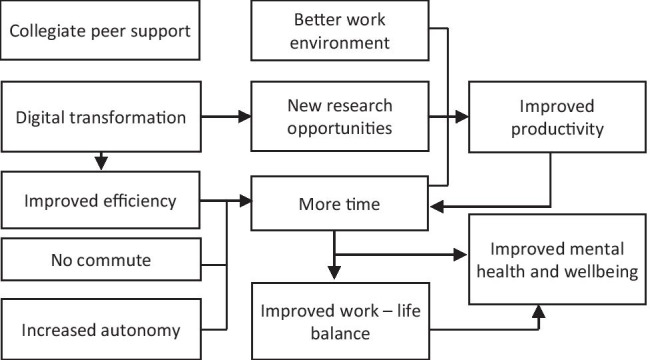
Benefits of lockdown reported by doctoral and early careers researchers

Benefits were only mentioned by half of the respondents, with this figure lower (40%) among those with caring responsibilities. The benefits identified were structured into 11 themes (Fig. 2). Although concerns surrounding time were a challenge for many, a benefit for others was having *more time*, which appeared to arise for several reasons. Some explained that *more time* stemmed from interruptions to their research: “Closure of the lab has meant I have fewer tasks to juggle simultaneously, allowing me to allocate more time to the work I can still do remotely” (DR, STEM). For many, working from home resulted in them having *no commute*, enabling them to redirect time previously spent in transit to their workplace:

I no longer have to spend hours commuting to and from work and so this time can be spent on research. (ECR, SS)

Without having to commute, and with social distancing regulations in place that keeps me mostly at home, I have found there is more time in the day to work. I’ve found that I can shift my working schedule to start earlier in the morning, which suits me as a ‘morning person’. (ECR, MS)

As reflected in this quotation, the more flexible home-working arrangements also *increased autonomy*, enabling researchers to choose their working approach and affording some additional time to think, read, focus, and reassess priorities:

As my day-to-day work can now be done at a more flexible time (I’m a Lecturer so I can pre-record lectures for my students). I may find I can get some more chunks of free time to write my thesis. (DR, MS)

Some also reported *improved efficiency* in working practices, some of which was due to the *digital transformation*, with the shift to online meetings and research seminars reducing the time required to attend such events. Other also felt meetings were more productive:

The online virtual research events and meetings are much easier to attend and also are recorded so that you can watch back if you cannot attend, meaning that research events and meetings are more accessible. (ECR, SS)

Relatedly, some individuals, including disabled people, identified that the new digital opportunities made it easier for them to engage equitably:

As a disabled person, the move to virtual connectivity has been what I have been asking and hoping for, for years. I now have more choice in what I attend, more flexibility and it feels more inclusive for someone like me. (ECR, SS)

Related to the shift to working online, many researchers benefited from *new research opportunities.* Many felt it was easier to connect and collaborate, with new, affordable, and more efficient access to international colleagues and events:

Potentially more international collaborations; meetings with colleagues made easier as no geographic boundaries online. (ECR, SS)

I’ve been able to focus on certain aspects of my work (writing) and have been able to work with like-minded colleagues to produce rapid, brief pieces of output. (ECR, MS)

Additionally, *new research opportunities* presented by the *digital transformation*, as well as the ongoing scientific needs of the pandemic, were highlighted. Arts and humanities researchers, for instance, commented on the benefits of digitisation of physical resources, which increased accessibility: “The National Archives making their digitised files available for free has been a positive and reduced my requirement to travel. Usually, they are too expensive for academic researchers to consider them as an alternative” (ECR, AH). Researchers across academic areas explained how the pandemic had created some *new research opportunities*, for example, by enabling research participants from a wider geographic area to take part online or by inspiring new, pandemic-related research directions:

It has triggered new ideas for research in my area caused by the outbreak. For example, COVID-19 consequences for the health system and primary care. (ECR, MS)

As my research is on crisis and disasters, the COVID-19 pandemic has provided a real-time case study. (DR, SS)

I am working on the COVID-19 pandemic, so I have more work. (ECR, STEM)

Working from home also provided some researchers with a *better work environment*, which supported *improved productivity*. Working from home presented some with an opportunity to work in a quieter environment, without distractions:

Working at home in pleasant, quiet, sunny surroundings, with no interruptions from kind friends who drop in, say ‘I hope I’m not interrupting’, accept a coffee and stay an hour and a quarter…The humane, relaxed sequestration is actually ideal for thinking out and writing the results of research. (ECR, STEM)

Researchers who had the opportunity to capitalise on these *new research opportunities* commented on new ways to share their work, thus reflecting *improved productivity*: “There is also an increase in webinars for sport coaches (the end user of my research), so I have been able to disseminate some of my work” (ECR, SS). With *improved efficiency, no commute, increased autonomy,* and *more productivity* helping researchers to have *more time*, this contributed to *improved work-life balance****:***

I feel like I am more productive and get the same amount of work done in less time, which gives me more time to relax. I can always take a break whenever I need to and make up for it later in the day. (DR, AH)

Although childcare responsibilities were identified as a challenge for many, the benefits of spending more time with family due to working from home were also noted. Some researchers also remarked that their *improved work-life balance* allowed them to engage in more hobbies: “Less time spent commuting (9 h spread over 3 days), so I have more time to spend time with my children and to fit in online exercise classes that have been set up” (ECR, MS). With some now having *more time* and *improved work-life balance*, this was connected to *improved mental health and wellbeing.* Some felt life was now less stressful and more time could be dedicated towards self-care activities:

Not having to go into the lab has given me extra time to focus on regular exercise, my mental health, indoor hobbies and keeping in touch with friends I haven’t spoken to in a while, which I might not usually have the time for. (DR, STEM)

Echoing these sentiments, many hoped changes imposed by the pandemic would encourage universities to revise working arrangements and allow greater flexibility in future:

I hope there will be a small long-term benefit that universities and the people that work in them will realise the sorts of issues that have been raised recently (such as remote working, flexible working, the importance of time with children and so forth) have also been raised a lot over the years and that finally we will see some lasting recognition that care work is important work, that remote working is possible. (ECR, SS)

A few also described silver-linings, stating that despite lockdown challenges, this strengthened *collegiate peer support*, as captured by responses such as “Colleagues are more collaborative” (DR, AH), and “I’ve developed much closer working relationships with a few key people and this has felt very supportive” (ECR, MS).

## Discussion

This paper explored the experience of doctoral researchers and ECRs in the first national lockdown in the UK, providing insights into perceived challenges and benefits arising as a result of changes imposed by the pandemic. Drawing on data from a large sample of doctoral researchers and ECRs, our findings highlight the need for HE institutions and policymakers across the sector to consider the impact of the pandemic on a community who represent the future of research, teaching, and leadership across the HE sector. Challenges arising as a result of the lockdown were more frequently reported than benefits. Echoing wider concerns across the sector (Watermeyer et al., 2021; Wray & Kinman, 2021), many respondents identified feeling frustrated by poor leadership and management through the early stages of the pandemic. While the findings demonstrate that the lockdown presented challenges for almost all participants, a range of benefits were also generated, which should be considered as the sector continues to navigate through the pandemic and comes to grips with its effects. Building on the quantitative results from the larger sample, which reported low levels of mental wellbeing and high levels of psychological distress (Byrom, 2020), our qualitative findings highlighted the complex interplay between the personal, professional, and educational circumstances that may have led to these outcomes. We focus our discussion on findings that could have long-term implications for policy and practice for doctoral researchers and ECRs.

### Working from home in a pandemic

Working from home blurred boundaries between the professional and personal lives for many respondents. For researchers with children, the challenges faced while working from home were compounded by additional home-schooling and caring responsibilities. The lockdown disintegrated boundaries between familial and professional realms, compromising the professional role of many researchers and placing more pressure on their ability to meet intensified work expectations. There is a real risk that the impact for researchers with caring responsibilities will be long-term and could stall career progression. Since the beginning of the pandemic, the challenge of disintegrated boundaries and the career implications of this have been greatest for those who identify as female and have caring responsibilities (e.g. Lerchenmüller et al., 2021; Myers et al., 2020; Ribarovska et al., 2021). Therefore, universities and research funders should consider issues of equity and fairness in career progression arising as a result of the pandemic. Evaluation and progression frameworks should be adapted to take into account the loss of productivity that is likely to have arisen for many researchers. With many HE institutions across the globe now engaging in initiatives to advance equity, diversity, and inclusion (e.g. Athena SWAN or ADVANCE—Rosser et al., 2019), it is vital that HE institutions re-double their commitment to address the disproportionate effects of the pandemic, to ensure that existing privileges and inequities are not reinforced.

While researchers with caring responsibilities emphasised the difficulties of working from home, the blurring of professional and personal realms was experienced more widely. Prior to the pandemic, some universities argued that researchers and academics could not work from home as this would present challenges for students (Kebritchi et al., 2017) and difficulties for roles and tasks that require a physical presence on campus (Smyth et al., 2021). However, over the last 18 months, the sector has demonstrated that remote working is feasible. Thus, beyond the pandemic, institutions should rethink their attitudes towards home-working and implement organisational policies that help researchers maintain autonomy and flexibility. It is paramount, however, that home-working is supported, not merely allowed. Attention should be directed towards helping researchers to establish and maintain work-life boundaries. How can a researcher be reassured that they have done enough and can afford to put their work away without potentially harming their career progression? This was a challenge prior to the pandemic (Metcalfe et al., 2018), and cross-sectional evidence indicates that doctoral researchers who identify with high levels of self-deprecation (i.e. imposter phenomenon) are more likely to report poor mental wellbeing (Byrom et al., 2020). Without serious attention, working from home could exacerbate the culture of long working hours (Sang et al., 2015) and increase the already concerning levels of burnout (Guthrie et al., 2017) in academia. Therefore, institutions should consider the management, mentoring, supervision, and training provided for doctoral researchers and ECRs, recognising the disruption that has been experienced and the additional challenges around boundaries and self-confidence that working from home evokes.

Institutions must also consider practical steps to help researchers create effective working environments at home. Working remotely requires researchers to have the space and resources to set up a viable office in their home. Many doctoral researchers live in rented accommodation with limited space, often using the same single room to sleep, work, and eat. While some researchers identified working from home as providing a better work environment, supporting more productivity, many reported a substantially different experience. The support that researchers have received to work from home is variable, raising questions about the universities’ responsibility to enable efficient and ergonomic home-working. Researchers pointed to the financial burden of setting up a home office. Universities need to consider their responsibility to reimburse or cover these costs, a solution that seems reasonable given the decreased overhead costs institutions might experience because of reduced operations on-campus (Mwando et al., 2021). Furthermore, supervisors, line managers, departments, and institutions must reflect upon current practices and consider how they demonstrate care (Noddings, 2013) to serve the unique needs and circumstances of this community.

### Working in isolation

The closure of university campuses resulted in researchers becoming physically disconnected from colleagues, supervisors, peers, and research groups, with many identifying a desire for contact with colleagues and peer support. Loneliness experienced through the lockdowns is not unique to the research community (Hwang et al., 2020; Killigore et al., 2020), but evidence of this is concerning given that mitigating isolation at the start of an academic career can be pivotal to career prospects (Belkhir et al., 2019), and that isolation is a prominent risk factor for poor mental health in doctoral researchers (Hazell et al., 2020; Metcalfe et al., 2018). If working from home is to be supported in the long-term, serious consideration must be given to protecting and promoting the collegiate relationships between researchers. Furthermore, HE institutions and key players across the HE sector (e.g. scholarly bodies, funders) should proactively take steps to direct resources towards the networking opportunities afforded to doctoral researchers and ECRs. The provision of such opportunities is paramount to ensure that researchers do not become increasingly isolated and/or miss out on some of the many potential benefits of research collaborations, such as the exchanging of ideas, development of new skills, access to funding, and production of higher quality outputs.

### Employment precarity and funding uncertainty

The last 18 months have been a difficult time financially for the HE sector, and many respondents voiced their concerns about careers and future employment as a result of measures—or the lack thereof—taken in response to the financial crisis. Concerns with careers and future employment are not new; as a community primarily working on highly competitive, short-term contracts, worries about the next career move are never far away (Byrom et al., 2020; Metcalfe et al., 2018). However, the pandemic has accentuated this, creating unease about the time and opportunities lost. Despite realising some small savings on staff overheads during the pandemic, universities in the UK have experienced substantial financial losses due to the reduction of income from accommodation, catering, and student recruitment, alongside investments to ensure campus buildings are COVID-19-secure (Burki, 2020). Many fixed-term contracts are not being renewed and recruitment suspensions have been implemented (Watermeyer et al., 2021). This contraction will have real implications for the doctoral and early career research community, heightening job precarity and aggravating funding uncertainty. Finding ways to minimise the impact of the pandemic on the career prospects of doctoral researchers and ECRs will be key to reducing concerns in this community.

### Limitations and future directions

The findings should be interpreted in light of several limitations. Our analysis is based on the perspectives of doctoral researchers and ECRs in the UK during the first national lockdown. Given that substantial changes have occurred in the sector since then, it remains unknown how the experiences of this community are similar or different to those at later stages in the pandemic. However, as pointed out in our discussion, many of the consequences of the initial lockdown could have longer-term effects for doctoral researchers and ECRs if intervention measures are not put in place. Despite this, there is a need to investigate the long-term impact of working from home and changes that have arisen because of the pandemic for these groups. Although the findings may resonate with the experiences of doctoral researchers and ECRs in the UK, therefore potentially achieving naturalistic generalisability for some, they may not resonate with the experience of researchers in other countries given that the nature, degree, and longevity of the pandemic’s impact on HE have varied internationally; the findings should, however, be of interest internationally given the UK’s large contribution to the international scientific community.

## Conclusions

Although disruption caused by the pandemic has demonstrated benefits in that working from home may now be more feasible for some researchers, the significant challenges posed could have a long-lasting impact on the career prospects of doctoral researchers and ECRs. Support from HE institutions is integral to creating stability and success for this community beyond the pandemic. Universities should lead the way in addressing longstanding challenges in this population by grasping this unique opportunity to protect and support the futures of the next generation of researchers, teaching staff, and academic leaders.

## Footnotes

1 Participants completed validated measures of psychological distress, loneliness, and mental wellbeing, and ratings of institutional and supervisory support.
